# The structure of a tautomerase superfamily member linked to the type VI secretion system of *Acinetobacter baumannii*

**DOI:** 10.1107/S2053230X22011414

**Published:** 2023-01-01

**Authors:** Genady Pankov, Gabriela Mol Avelar, Grant Buchanan, Sarah J. Coulthurst, William N. Hunter

**Affiliations:** a Division of Biological Chemistry and Drug Discovery, School of Life Sciences, University of Dundee, Dundee DD1 5EH, United Kingdom; b Division of Molecular Microbiology, School of Life Sciences, University of Dundee, Dundee DD1 5EH, United Kingdom; Centre for Cellular and Molecular Biology, Hyderabad, India

**Keywords:** *Acinetobacter baumannii*, 4-oxalocrotonate tautomerase, tautomerase superfamily, type VI secretion system

## Abstract

Bacteria exploit specialized secretion systems to assist in competition for resources, in collaboration and in communication. Here, a protocol for the recombinant production, purification and crystallization of a protein linked to the *Acinetobacter baumannii* type VI secretion system is provided. A high-resolution structure of this trimeric protein is reported, revealing the characteristic dual β–α–β subunit fold typical of longer subunit members of the tautomerase superfamily. The protein does not appear to be toxic to bacteria or yeast under the conditions tested. The possible biological role of this protein is discussed.

## Abstract 2

The high-resolution crystal structure of an *Acinetobacter baumannii* protein previously reported as being dependent on the type VI secretion system has been determined. Structural and sequence comparisons reveal significant conservation with the tautomerase superfamily of enzymes, which are not generally noted as being associated with this secretion system. The protein does not display toxicity when produced in either a bacterial or a yeast system. The implications of these observations are discussed.

## 1. Introduction

The type VI secretion system (T6SS) is a specialized secretion apparatus used by a subset of Gram-negative bacteria to gain advantage in competition and/or to help establish a replicative niche (Coulthurst, 2019 ▸). Much of the research into the T6SS has focused on aspects of interbacterial competition, although it is now recognized as a system that also contributes to interactions between some Gram-negative bacteria and microbial eukaryotes in addition to its originally described role against host eukaryotic organisms (Hernandez *et al.*, 2020 ▸; Robitaille *et al.*, 2021 ▸; Trunk *et al.*, 2018 ▸, 2019 ▸). The T6SS secretes protein cargo either out to the environment or into a competitor, the prey, to elicit an effect. Previous studies have identified that these cargos or effectors are primarily destructive enzymes, in effect toxins, that compromise the existence of competitors. When involved in competition the bacteria need to protect themselves from self-intoxication by antibacterial effectors, and to do so will co-express genes encoding cognate immunity proteins that are able to neutral­ize endogenous and exogenous toxins. The absence of the gene encoding an immunity protein adjacent to an effector-encoding gene might indicate that the effector is not directly involved in interbacterial competition. It is now recognized that the T6SS is a highly versatile tool that facilitates competition. These systems involve multiple families of peptidoglycan hydrolases, phospholipases, nucleases, cofactor hydrolases and effectors which form pores in bacterial membranes in addition to modulating diverse intracellular and extracellular processes such as adhesion, internalization, cytoskeletal assembly, evasion of host immune responses and even nutrient scavenging (Monjarás Feria & Valvano, 2020 ▸; Yang *et al.*, 2021 ▸).

*Acinetobacter baumannii* is an important nosocomial pathogen that exploits the T6SS (Harding *et al.*, 2018 ▸; Le *et al.*, 2021 ▸). Analysis of the genome of *A. baumannii* strain AB307-0294 revealed the presence of genes encoding the T6SS machinery, whilst further genomics analysis identified in excess of 240 individual candidate T6SS-associated effectors in 23 strains. These included putative lipases, nucleases, peptidoglycan hydrolases and deaminases. Subsequent analysis of proteins that are secreted in a T6SS-dependent manner by AB307-0294 confirmed the assignment of a number of these candidates and further identified several other proteins potentially representing substrates for this secretion apparatus (Fitzsimons *et al.*, 2018 ▸).

Of interest to us was the identification of a protein, encoded by the gene ABBFA_002011, in the AB304-0294 secretome (Fitzsimons *et al.*, 2018 ▸). The encoded protein, which is classed as a member of the tautomerase superfamily on the basis of sequence homology, represents a putative effector. The absence of any apparent immunity partner was noted. This is the only example, to the best of our knowledge, of a putative tautomerase superfamily member linked to a T6SS. We labelled the protein *Ab*TFM (*A. baumannii* tautomerase family member) and sought to investigate further. The tautomerase superfamily is promiscuous in terms of substrate specificity and provides examples of diverse enzyme activity such as isomerases, dehalogenases, decarboxylases and hydratases (Poelarends *et al.*, 2008 ▸; Baas *et al.*, 2013 ▸; Huddleston *et al.*, 2014 ▸). Also included in the superfamily is the lymphokine called macrophage inhibitory factor that is involved in immune response regulation (Huddleston *et al.*, 2014 ▸).

Here, we provide protocols for the recombinant production, purification and crystallization of *Ab*TFM. We show that our construct for *Ab*TFM does not have any deleterious effect when produced in recombinant form in a bacterium, *Escherichia coli*, or a yeast, *Saccharomyces cerevisiae*. We report structural details and comparisons with bacterial homologues and speculate about the potential biological function of the protein.

## 2. Materials and methods

### 2.1. Protein production

A synthetic gene encoding *Ab*TFM (UniProt ID B0VC61; The Uniprot Consortium, 2021 ▸), optimized for expression in *E. coli*, was ordered (Twist Biosciences) and subcloned into the expression vector pET-15b-TEV (modified from pET-15b, Novagen). The resulting plasmid produces protein with an N-terminal hexahistidine tag that is cleavable by Tobacco etch virus (TEV) protease. The plasmid was introduced into *E. coli* BL21 (DE3) cells by heat-shock transformation for the production of recombinant protein. The bacteria were cultured at 310 K (37°C) to an optical density at 600 nm of 0.5–0.6 in Luria–Bertani medium containing 50 mg l^−1^ carbenicillin. Gene expression was induced with isopropyl β-d-1-thiogalactopyranoside (final concentration 1 m*M*) and growth of the culture continued for approximately 16 h at 295 K (22°C) prior to harvesting the cells by centrifugation (4200*g *for 30 min at 277 K (4°C). The buffers employed for *Ab*TFM purification were buffer *A* (50 m*M* HEPES–NaOH pH 6.8, 250 m*M* NaCl) and buffer *B* (buffer *A* with 0.5 *M* imidazole). Cells in buffer *A* were lysed using a Cell Disrupter (Constant Systems) at 207 MPa and the lysate was clarified by centrifugation at 40 000*g* for 30 min at 277 K (4°C). The supernatant was filtered (0.2 µm) and loaded onto a 5 ml HisTrap HP column (GE Healthcare) previously charged with Ni^2+^ for an initial affinity-chromatography step. The target protein eluted between concentrations of 180 and 250 m*M* imidazole (using buffer *B*). Fractions containing *Ab*TFM were pooled and treated with His-tagged TEV protease (1 mg of protease per 10 mg of protein) overnight at 277 K (4°C). The mixture of *Ab*TFM, TEV protease and cleaved His tag was then dialyzed against buffer *A* to remove excess imidazole. This was followed by reverse affinity chromatography on the HisTrap column to remove the protease, the tag and any *Ab*TFM that retained a tag. A final purification step was achieved by size-exclusion chromatography (SEC) on a calibrated Superdex 75 16/60 gel-filtration column in 20 m*M* HEPES–NaOH pH 6.8, 150 m*M* NaCl. The theoretical mass of the *Ab*TFM poly­peptide is approximately 15.5 kDa. The SEC profile was well defined and suggested the presence of a single multimeric species of approximate molecular mass 67 kDa. Fractions containing *Ab*TFM were pooled and concentrated using centrifugal force (Sartorius). The high level of sample purity was confirmed by sodium dodecyl sulfate–polyacrylamide gel electrophoresis using stain-free gels (Bio-Rad) prior to crystallization trials. The approximate yield of *Ab*TFM was 4 mg per litre of *E. coli* culture. Protein concentrations were determined from the absorbance at 280 nm measured using a DeNovix spectrophotometer (*A*_280_; Cambridge Bioscience). Concentrations were determined from the Beer–Lambert law using the predicted molar extinction coefficient (ɛ = 14 440 *M* ^−1^ cm^−1^ at 280 nm) obtained from *ProtParam* (Gasteiger *et al.*, 2005 ▸).

### 2.2. Crystallization

A preliminary crystallization screen was performed using the sitting-drop vapour-diffusion method in which 0.2 µl *Ab*TFM at 2 mg ml^−1^ and 0.2 µl reservoir solution were equilibrated over 50 µl reservoir solution in 96-well plates at 296 K (23°C). Commercial crystal screens from Qiagen (The Classics Suite and AmSO4 Suite), Molecular Dimensions (JCSG-plus and Morpheus) and Hampton Research (PEGRx) were used. Prism-shaped crystals, typically with dimensions of 10 × 15 × 20 µm, were observed after five days using condition C3 of the JCSG-plus screen consisting of 0.2 *M* ammonium nitrate, 20%(*w*/*v*) PEG 3350. The shape and dimensions of these crystals were optimized by increasing the volume of the drop and the protein concentration and by microseeding (Stura & Wilson, 1990 ▸). For microseeding, a slurry of crystals was transferred to a microcentrifugation tube containing mother liquor and a small nylon ball (Hampton Research). The mixture was agitated to fragment the crystals, creating a stock mixture (Luft & DeTitta, 1999 ▸). Serial dilutions of this stock were prepared, and a nylon loop was passed through these and then dipped into the crystallization drops. Well formed crystals, typically with dimensions of 60 × 60 × 80 µm, grew over 24 h at 296 K (23°C) in a sitting drop consisting of 1 µl *Ab*TFM at 5 mg ml^−1^ (in 20 m*M* HEPES–NaOH pH 6.8, 150 m*M* NaCl) and 1 µl reservoir solution [0.2 *M* ammonium nitrate, 18%(*w*/*v*) PEG 3350]. Crystallization information is summarized in Table 1 ▸.

### 2.3. X-ray data collection and processing, structure determination and refinement

Crystals were cryoprotected in Paratone-N and then flash-cooled in liquid nitrogen prior to X-ray exposure. After testing several crystals, diffraction data were subsequently collected from a single specimen using a Rigaku MicroMax-007 HF rotating-anode X-ray generator equipped with a Saturn 944HG1 CCD detector. Data to 1.70 Å resolution were integrated using *iMosflm* (Battye *et al.*, 2011 ▸) and then processed with *AIMLESS* (Evans, 2011 ▸) from the *CCP*4 suite (Winn *et al.*, 2011 ▸). The crystal belonged to space group *H*3, with unit-cell parameters *a* = 59.185, *b* = 59.185, *c* = 97.757 Å, α = β = 90.0, γ = 120.0°. The Matthews coefficient (Matthews, 1968 ▸) suggested one monomer in the asymmetric unit (*V*_M_ = 2.15 Å^3^ Da^−1^ and about 43% bulk solvent) and consideration of the space-group symmetry together with SEC data suggested the presence of a homotrimer.

A single subunit of the hexameric putative 4-oxalo­crotonate tautomerase from *Nostoc* sp. (PDB entry 4lkb, 2.16 Å resolution, space group *H*3, 48% sequence identity; D. Kumaran, S. C. Almo & S. Swaminathan, unpublished work) provided a model for molecular-replacement calculations in *Phaser* (McCoy *et al.*, 2007 ▸). The structure from *Nostoc* sp. was first modified in *CHAINSAW* (Stein, 2008 ▸) by pruning non­conserved residues to a γ atom (62 residues were altered and 58 remained conserved) and the solvent molecules were removed. The polypeptide was positioned in the unit cell and the resulting electron-density and difference density maps allowed the sequence of *Ab*TFM to be assigned and a new model to be built using *Coot* (Emsley *et al.*, 2010 ▸). Several rounds of electron-density and difference density map inspection, model manipulation and refinement in *REFMAC*5 (Murshudov *et al.*, 2011 ▸) led to the incorporation of solvent, chloride and nitrate ions, and several dual rotamers. *MolProbity* (Chen *et al.*, 2010 ▸) was used at different stages to monitor model geometry in combination with the validation tools provided in *Coot*. Structural and sequence comparisons of *Ab*TFM with orthologous structures in the PDB was carried out using *DALI* (Holm, 2020 ▸) and *XtalPred* (Slabinski *et al.*, 2007 ▸). Molecular images were rendered using the *PyMOL* graphics system (Schrödinger). Crystallographic statistics are presented in Tables 2 ▸ and 3 ▸.

### 2.4. Toxicity assay

To observe the impact of heterologous expression in *E. coli*, genes encoding *Ab*TFM or *Ab*TFM fused with an N-terminal Tat signal peptide (from *Serratia marcescens* SufI) were cloned into the arabinose-inducible expression vector pBAD18-Kn (Guzman *et al.*, 1995 ▸) and expressed in *E. coli* MG1655 cells. Freshly transformed *E. coli* MG1655 cells grown overnight on solid medium were resuspended in LB, adjusted to an OD_600_ of 1 and serially diluted, and 5 µl was spotted onto LB agar containing 100 µg ml^−1^ kanamycin and 0.2% glucose, 0.02% l-arabinose or 0.2% l-arabinose, followed by incubation at 310 K (37°C) overnight. To observe the impact of *Ab*TFM expression in *S. cerevisiae*, the gene encoding *Ab*TFM was cloned into the galactose-inducible expression vector pRB1438 and transformed into *S. cerevisiae* K699 cells as described by Trunk *et al.* (2018 ▸). Transformed *S. cerevisiae* K699 cells were pre-grown in DOA-URA (Merck) plus 2% raffinose liquid medium at 303 K (30°C), adjusted to an OD_600_ of 1 and serially diluted, and 5 µl was spotted onto DOA-URA plus 2% raffinose agar containing 1% glucose, 0.5% galactose or 1% galactose, followed by incubation at 303 K (30°C) for four days.

## 3. Results and discussion

### 3.1. The fold and quaternary structure of *Ab*TFM

**Figure 1:**
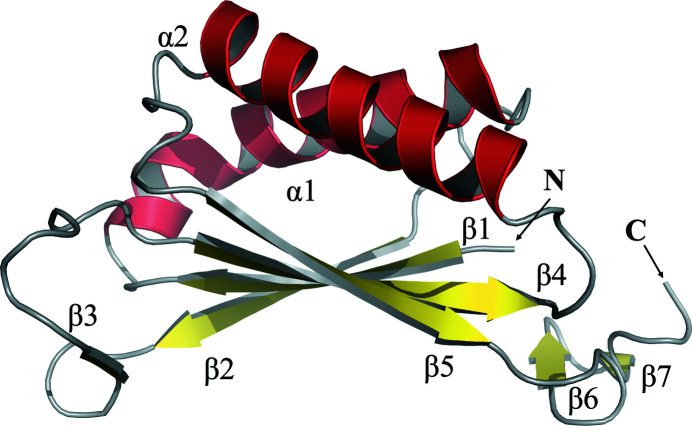
The secondary structure of an *Ab*TFM subunit. Helices are shown in red, sheets in yellow and loops in grey. Sheets and helices are numbered consecutively from the N- to C-termini, which are labelled.

**Figure 2:**
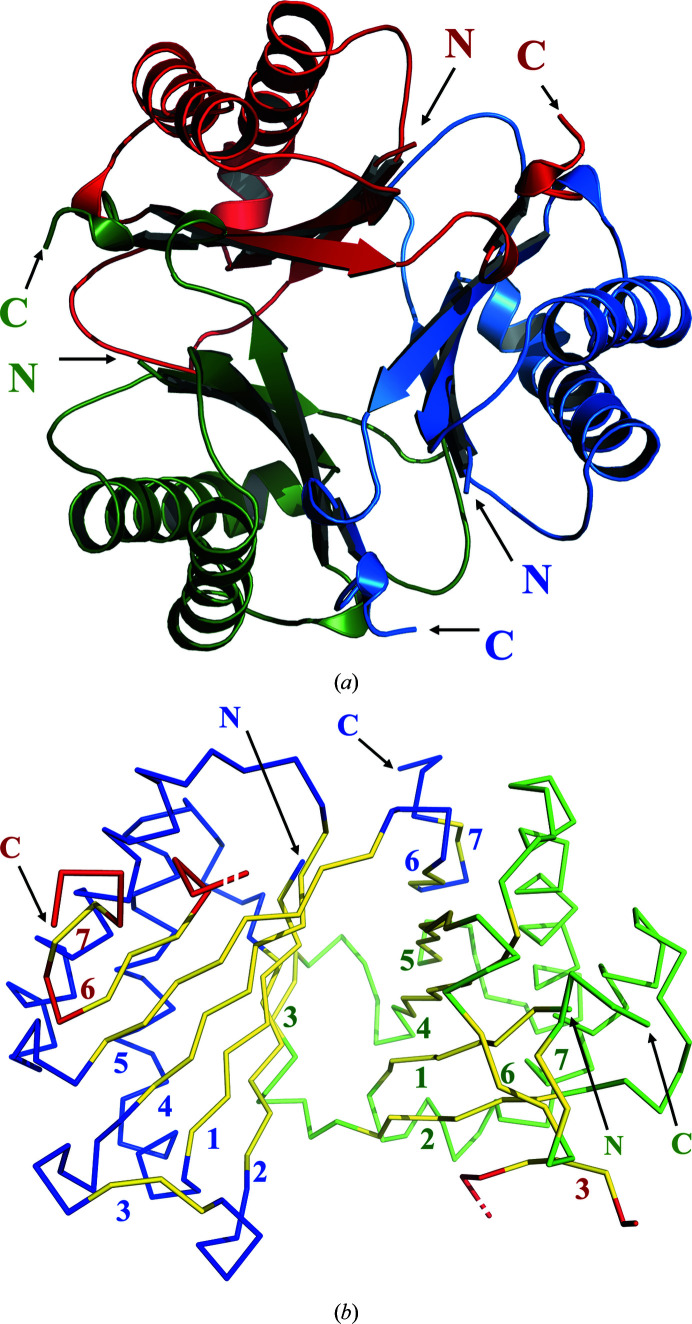
The *Ab*TFM assembly. (*a*) A ribbon diagram of three subunits viewed down the threefold axis of symmetry. Chains *A*, *B* and *C* are shown in green, blue and red, respectively, with the N- and C-termini labelled. (*b*) A C^α^ trace of two subunits, *A* (green and yellow) and *B* (blue and yellow), and parts of subunit *C* (red and yellow) to show two of the seven-stranded sheets that help to create the trimeric assembly. The two strands are coloured yellow and are numbered. The numbers are coloured according to the assigned subunit.

The structure of *Ab*TFM, with a single subunit in the asymmetric unit, was determined by molecular replacement and refined using diffraction data extending to 1.75 Å resolution. Although the data-collection statistics (Table 3 ▸) suggest that higher resolution data could likely be obtained, we considered this data set to be of a suitable quality to provide the structural insight that we sought. The resulting model consists of residues Met1–Leu121. Due to the lack of reliable electron density, the first two residues (Gly-His), which are remnants of the TEV protease cleavage site, and the last eight (Leu-Leu-Asn-Tyr-Gln-Val-Asn-Ile) residues are absent. The compact fold of the *Ab*TFM subunit, in which about 75% of the residues are in elements of secondary structure, displays the characteristic β–α–β repeat structure of the longer tautomerase superfamily (Whitman, 2002 ▸; Fig. 1 ▸). This forms a subunit structure with two α-helices on one side and four strands aligned in order β2–β1–β4–β5 on the other to form the core of a β-sheet. The strands are paired so that 1 and 2 and then 4 and 5 are parallel but the pairs are antiparallel to each other. There are three other short β-strands: β3 occurs in the loop linking β2 and β4, and β6 and β7 then form an antiparallel alignment at the C-terminal end of the polypeptide. *Ab*TFM forms a homotrimer exploiting a crystallographic threefold axis of symmetry parallel to *c* (Fig. 2 ▸). Extensions to the core β-sheet are noted, whereby on one side β3 from a partner subunit is aligned antiparallel to β2 and on the other β6 and β7 from the remaining subunit extend the sheet. The interactions between elements of secondary structure on each subunit to form three seven-stranded β-sheets are important for trimer formation.

**Figure 3:**
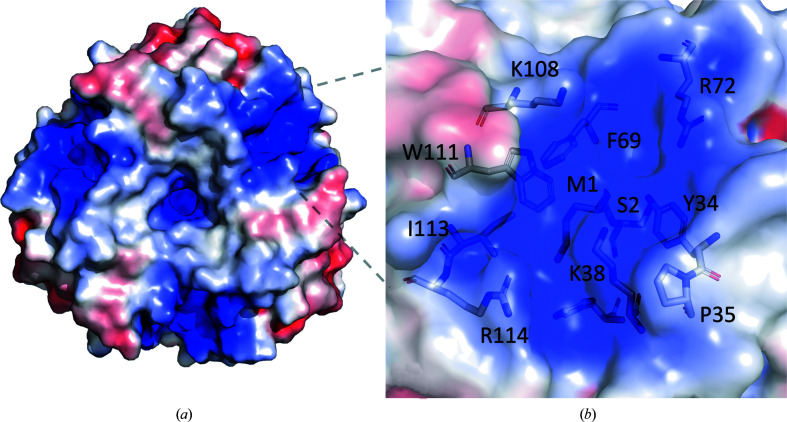
(*a*) The surface of the *Ab*TFM trimer, viewed down the threefold axis of symmetry, coloured according to electrostatic potential from positively charged in blue to negatively charged in red. (*b*) A close-up of one potential binding site. Residues that help to form the binding site are shown as sticks.

**Figure 4:**
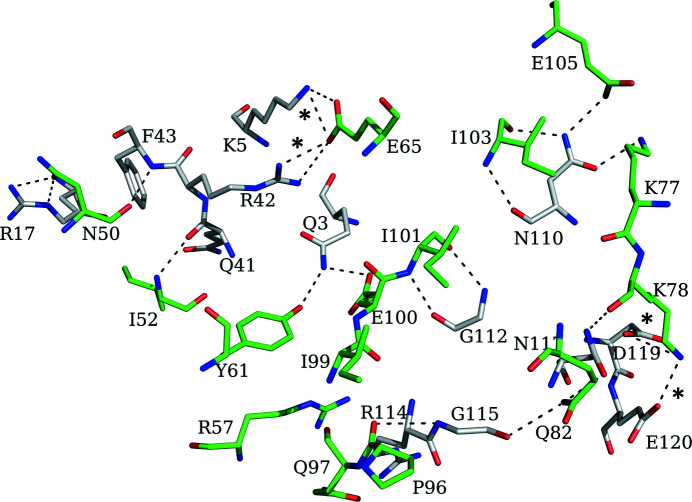
Residues involved in formation of the protein–protein interface between two subunits of *Ab*TFM. The C atoms of different subunits are coloured green and grey. Asterisks indicate the position of salt-bridge interactions

The interface between each monomer creates three identical pockets lined with positively charged side chains. The shape and the charge of these pockets would be consistent with *Ab*TFM binding a small acidic entity (Figs. 3 ▸ *a* and 3 ▸ *b*). Analysis of the protein–protein interactions involved in the formation of the oligomer identified four salt bridges per interface: Lys78–Asp119, Lys78–Glu120, Glu65–Lys5 and Glu65–Arg42 (Fig. 4 ▸). In addition, there are inter-subunit hydrogen bonds involving both main-chain and side-chain groups (Fig. 4 ▸), together with van der Waals interactions to stabilize the trimeric assembly. According to *PISA* (Krissinel & Henrick, 2007 ▸), the accessible surface areas (ASA) of the *Ab*TFM subunit and homotrimer are approximately 7440 and 19 630 Å^2^, respectively. This indicates that an ASA of about 2700 Å^2^ is utilized in oligomerization. This is almost one third of the subunit surface area and is indicative of a highly stable quaternary structure.

Members of the tautomerase superfamily form distinctive trimers or hexamers (Whitman, 2002 ▸) and the crystal structure indicates that *Ab*TFM forms a stable assembly that belongs to the trimer group. The mass of such a quaternary structure is approximately 46.5 kDa. The trimer presents three putative active or binding sites separated by approximately 25 Å. Each site is primarily constructed within a single subunit, with a combination of primarily nonpolar side chains and basic residues, in a relatively small depression formed between the β-sheet and the two helices. Oligomerization, and the presence of an adjacent subunit, contributes to the organization of one area of the active site. The position of the side chain of Phe40 of one subunit is supported by van der Waals packing against Pro54 on β3 of a partner subunit. This partner subunit also contributes the side chains of Arg57 and Tyr61 and a buried Glu100 to define the structure.

### 3.2. Comparisons with orthologues

A structural comparison, using the *DALI* server, of *Ab*TFM with orthologous structures identified that the model that was used for molecular replacement is the most similar structure. Superposition of the *Ab*TFM subunit on the six subunits of the putative 4-oxalocrotonate tautomerase from *Nostoc* sp. (PDB entry 4lkb, 48% sequence identity) gave *Z*-scores of around 12 and r.m.s.d. values of about 0.9 Å for overlays of 121 C^α^ positions. The next closest structure was a hypothetical protein from *Haemophilus influenzae* (PDB entry 1mww, 38% sequence identity; C. Lehmann, S. Pullalarevu, W. Krajewski, A. Galkin, A. Howard & O. Herzberg, unpublished work). Here, a *Z*-score of 11 and an r.m.s.d. of 1.04 Å were observed for overlays of 118 C^α^ positions. The third ‘relative’ is a malonate semialdehyde decarboxylase from *Pseudomonas pavonaceae* (PDB entry 2aag, 21% sequence identity; Almrud *et al.*, 2005 ▸), with *Z*-scores of about 9 and r.m.s.d. values of 1.5–1.7 Å for alignment of between 112 and 116 C^α^ positions for different subunits. This enzyme has proven decarboxylase and hydratase activities, and although it is a structural orthologue two factors suggest that the enzyme activities might be distinct for *Ab*TFM. The use of site-directed mutagenesis to make active-site substitutions in the *P. pavonaceae* enzyme identified that Asp37 and Arg73 are important for the decarboxylase and hydratase activities. The corresponding residues in *Ab*TFM are Lys38 and Ala70. This, together with differences in the shape of the active site, suggest that they are likely to bind different substrates.

**Figure 5:**
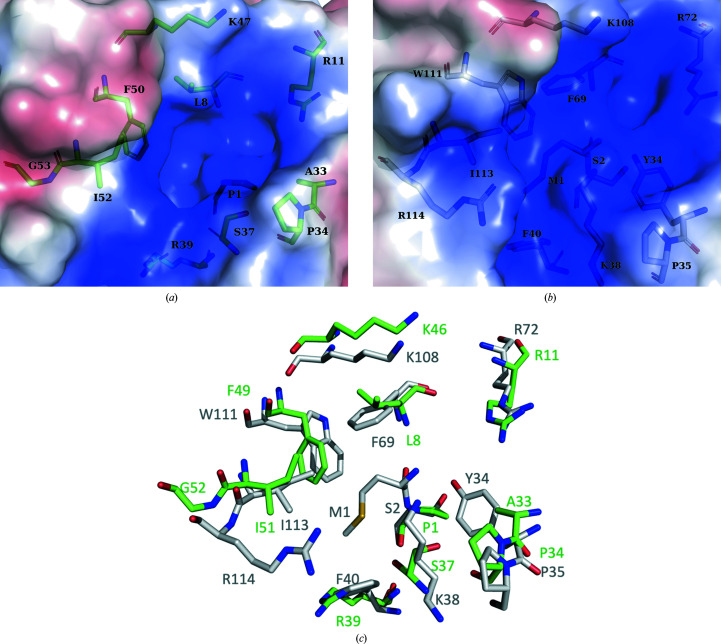
(*a*) The residues in the active site of 4-oxalocrotonate tautomerase with the protein surface shown as van der Waals radii and coloured red for acidic charge and blue for basic (PDB entry 1bjp). (*b*) The potential binding site of *Ab*TFM with the same depiction. (*c*) Superposition of the binding-site residues of 4-oxalocrotonate tautomerase and *Ab*TFM.

An overlay of a bone fide 4-oxalocrotonate tautomerase (Taylor *et al.*, 1998 ▸; PDB entry 1bjp) with *Ab*TFM was carried out (Fig. 5 ▸). This structure was chosen for comparison because it contains a covalent adduct to the Pro1 catalytic residue. The overlay places Pro1 N, a key functional group for catalysis, at distances of 1.7 Å from Ser2 N of *Ab*TFM and 3.7 Å from Met1 N. In our study we used recombinant *Ab*TFM that had an N-terminal tag to assist purification. Although the tag was cleaved by TEV protease, an N-terminal extension remains that would prevent the removal of the initiating methionine from the wild-type protein. There are numerous differences around the catalytic centre that suggest that the two proteins would process different substrates or bind different ligands. In *Ab*TFM, for example, there are four aromatic residues (Tyr34, Phe40, Phe69 and Trp111) and two basic residues (Lys38 and Arg72) that surround the N-terminus. The corresponding residues in *P. pavonaceae* 4-oxalocrotonate tautomerase are Ala33, Arg39, Leu8 and Phe50 (the latter two from a different subunit) and Ser37 and Arg11 (also from a different subunit).

### 3.3. *Ab*TFM does not display toxic effects under the conditions tested

**Figure 6:**
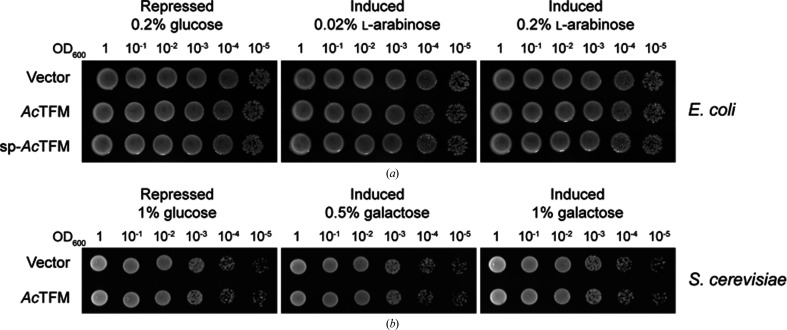
The *Ab*TFM protein does not display toxicity upon heterologous expression in bacterial or yeast cells. (*a*) Growth of *E. coli* MG1655 carrying an empty vector control (pBAD18-Kn; vector) or plasmids directing the expression of native *Ab*TFM (*Ab*TFM) or of *Ab*TFM with an N-terminal Tat signal peptide (sp-*Ab*TFM) on LB medium with 0.2% glucose or 0.02% or 0.2% l-arabinose to repress (glucose) or induce (l-arabinose) gene expression. (*b*) Growth of *S. cerevisiae* K699 transformed with an empty vector control (pRB1438; vector) or an integrative plasmid directing the expression of *Ab*TFM (*Ab*TFM) on DOA-URA plus 2% raffinose medium with 1% glucose or 0.5% or 1% galactose to repress (glucose) or induce (galactose) gene expression.

In order to determine whether *Ab*TFM is toxic to bacterial or fungal cells, as would be predicted if it were an antibacterial or antifungal effector, respectively, we adopted approaches used previously for other effectors (Fritsch *et al.*, 2013 ▸; Trunk *et al.*, 2018 ▸). The protein was produced heterologously in *E. coli* MG1655 and *S. cerevisiae* K699 cells (Fig. 6 ▸). In *E. coli*, *Ab*TFM was expressed in its native form (*i.e.* with no modification at the N-terminus), where it would remain localized in the cytoplasm, and also with an N-terminal Tat signal peptide which would direct it to the periplasmic compartment, since many T6SS antibacterial effectors have a target within the periplasm and only show significant toxicity in this compartment. Following induction of *Ab*TFM expression, there was no observable difference in the growth of *E. coli* or *S. cerevisiae* compared with non-induced conditions or an empty vector control (Fig. 6 ▸). This suggests that *Ab*TFM is unlikely to be a typical antibacterial or anti-eukaryotic T6SS effector protein.

### 3.4. What is the role of *Ab*TFM?

Our interest in *Ab*TFM was stimulated by its identification as a T6SS-dependent secreted protein (Fitzsimons *et al.*, 2018 ▸). This protein appeared to be unique, being the first example of a putative tautomerase linked to the T6SS. We investigated whether the protein might display T6SS-mediated toxicity consistent with that noted for antibacterial or anti-eukaryotic effectors. Our construct does not display toxic effects when produced in *E. coli* or *S. cerevisiae* (Fig. 6 ▸), suggesting that the protein is not an effector of the type commonly noted with the T6SS.

We investigated the genomic position of the gene encoding *Ab*TFM to determine whether a clue to its function might be found there. The context of the gene was examined in publicly available whole-genome sequences of multiple strains of *A. baumannii* using the PATRIC online resource (Davis *et al.*, 2020 ▸). We noted that the gene of interest is not within any T6SS operon and there is no obvious gene encoding an immunity protein nearby. The gene is placed downstream of two genes encoding components of transporter subunits and upstream of first a gene encoding a formate-dependent phosphoribosylglycinamide formyltransferse and then a gene encoding a potential bifunctional enzyme uridyltransferase/uridylyl-removing enzyme. The third downstream gene encodes succinyldiaminopimelate transaminase. These three enzymes are involved in purine metabolism, nitrogen assimilation and lysine biosynthesis, respectively, with no clear biological link. Intriguingly, the latter two enzymes are regulated by or use 2-oxoglutarate as a substrate, a small anionic compound that is similar to 4-oxalocrotonate in terms of size and polarity.

The structural data support the assignment of the protein as a member of the tautomerase superfamily of enzymes; moreover, if indeed it is an enzyme then it will utilize a small, highly polar acidic substrate. However, the lack of the ubiquitous N-terminal proline suggests that *Ab*TFM is not likely to be a 4-oxalocrotonate tautomerase. As an aside, we would point out that the structure we used as our starting model (PDB entry 4lkb) also lacks the N-terminal proline and the assignment of this protein as a putative 4-oxalocrotonate tautomerase should be treated with caution.

There are therefore a number of possible roles that T6SS-mediated secretion of *Ab*TFM may contribute to. The protein may be involved in a catabolic pathway, as are the majority of the superfamily members, where the function may be the provision of materials required for nutrient acquisition, for example. In addition, since *Acinetobacter* utilizes small polar lactones in quorum sensing (Chan *et al.*, 2011 ▸), we recognize the possibility that *Ab*TFM might participate in signalling pathways, processing extracellular substrates to generate or alter levels of quorum sensing or quenching agents. This might therefore represent an extension or adaptation of the system noted in *Vibrio cholerae* whereby quorum sensing contributes a role to control the expression of a T6SS with a link to virulence (Zheng *et al.*, 2010 ▸). Intriguingly, enzyme-like proteins have been identified as providing T6SS-dependent immunity proteins (Lopez *et al.*, 2021 ▸) and the possibility exists that *Ab*TFM might represent an effector-interacting protein. Finally, we cannot rule out the possibility that *Ab*TFM indeed fulfils a role in a host or target cell. The toxicity assays represent a blunt live–die test and would not detect more subtle effects that might involve signalling processes, for example.

Key to understanding the biological role of *Ab*TFM and why it was identified as part of the AB307-0294 secretome will be the identification of its substrate or ligands. Research towards such a goal will be facilitated by the provision of robust protocols for the production and crystallization of recombinant *Ab*TFM and the high-resolution crystal structure.

## Supplementary Material

PDB reference: tautomerase family member, 7yxv

## Figures and Tables

**Table 1:** Crystallization conditions for *Ab*TFM

Method	Vapour diffusion
Plate type	Sitting drop (Hampton Research)
Temperature (K)	296
Protein concentration (mg ml^−1^)	5
Buffer composition of protein solution	20 m*M* HEPES–NaOH pH 6.8, 150 m*M* NaCl
Composition of reservoir solution	0.2 *M* NH_4_NO_3_, 20%(*w*/*v*) PEG 3350
Volume and ratio of drop	1 µl protein solution:1 µl reservoir
Volume of reservoir (µl)	500

**Table 2:** Diffraction data-collection parameters

Diffraction source	Rigaku MicroMax-007 HF X-ray generator
Wavelength (Å)	1.54178
Temperature (K)	100
Detector	Saturn 944HG1 CCD
Crystal-to-detector distance (mm)	50
Rotation range per image (°)	0.5
Total rotation range (°)	360

**Table 3:** Crystallographic and refinement statistics Values in parentheses are for the highest resolution shell.

Data collection	
Space group	*H*3
Subunits in asymmetric unit	1
*a*, *b*, *c* (Å)	59.17, 59.17, 97.74
α, β, γ (°)	90.00, 90.00, 120.00
Resolution range (Å)	24.78–1.75
No. of reflections	96446
Unique reflections	13698
Completeness (%)	97.3 (87.0)
*R*_merge_ †	0.044 (0.131)
Multiplicity	7.0 (1.5)
*R*_p.i.m._ ‡	0.023 (0.131)
〈*I*/σ(*I*)〉	27.0 (4.1)
Wilson *B* factor (Å^2^)	7.2
CC_1/2_	0.999 (0.951)
Refinement	
*R*_cryst_ §/*R*_free_ ¶ (%)	0.14/0.18
No. of reflections for *R*_cryst_/*R*_free_	13022/675
No. of protein residues	121
No. of 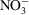 /Cl^−^ ions	1/1
No. of water molecules	135
Cruickshank DPI (Å)	0.098
R.m.s.d., bond lengths (Å)	0.013
R.m.s.d., angles (°)	1.682
Average *B* factors (Å^2^)	
Overall	12.5
Water molecules	25.0
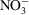	10.1
Cl^−^	28.3
Ramachandran analysis	
Residues in favoured regions (%)	98.3
Residues in allowed regions (%)	1.7
Rotamer outliers (%)	0

† *R*_merge_ = 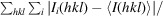 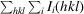 , where *I*_*i*_(*hkl*) is the intensity of the *i*th measurement of reflection *hkl* and 〈*I*(*hkl*)〉 is the mean value of *I*_*i*_(*hkl*) for all *i* measurements.

‡ *R*_p.i.m._ = 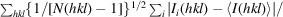 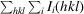 .

§ *R*_cryst_ = 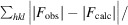 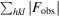 , where *F*_obs_ is the observed structure-factor amplitude and *F*_calc_ is the structure-factor amplitude calculated from the model.

¶ *R*_free_ is calculated using a subset of data that were excluded from refinement calculations (5%) using the same method as for *R*_merge_.
